# EXAFS and Rotating Disc Electrode Study into the Thermochromic Behavior of Nickel Salts in Deep Eutectic Solvents

**DOI:** 10.1021/acs.jpcc.5c05771

**Published:** 2025-11-11

**Authors:** Jennifer M. Hartley, George Tebbutt, Andrew Ballantyne, Charlotte Ashworth-Güth, Gero Frisch, Karl S. Ryder

**Affiliations:** † School of Chemistry, 4488 University of Leicester, Leicester LE1 7RH, U.K.; ‡ Institut für Anorganische Chemie, 26545 TU Bergakademie Freiberg, 09599 Freiberg, Germany; § School of Geology, University of Leicester, Leicester LE1 7RH, U.K.

## Abstract

The thermochromic and electrodeposition behavior of nickel chloride was investigated in two choline chloride-based deep eutectic solvents (DES), with either ethylene glycol or urea as the hydrogen bond donors. In the ethylene glycol DES, thermochromism was found to be reversible, with ligand exchange resulting in the main structural change from octahedral to tetrahedral coordination taking place between 90 and 100 °C. In the urea DES, a change in color only took place above 100 °C, at which point a suspected ammonia species was irreversibly formed from decomposition of the solvent. The speciation effects were studied by using UV–vis and EXAFS spectroscopies, together with the electrochemical methods of cyclic voltammetry and rotating disk voltammetry. The observed speciation changes evolving at higher temperatures were seen to correlate with more well-defined electrochemical behavior together with faster electron-transfer kinetics and higher Coulombic efficiency.

## Graphical abstract

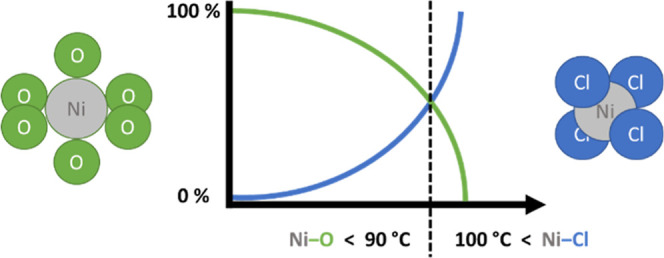

## 1 Introduction

Nickel plating is extensively used for corrosion resistance, fabrication of printed circuit boards, and decorative applications, with commercial plating formulations available from the early 1900s.^1^ However, plating baths have to be operated under stringent process control, as small differences in pH and formulation can manifest large changes in the properties of the electrodeposit, such as brightness, hardness, thickness, roughness, and ductility.^2^ During deposition, issues such as low current efficiency, hydrogen embrittlement, and surface passivation/oxidation can arise. To circumvent these issues, the use of nonaqueous ionic media has been explored.

The electrodeposition of nickel has been carried out in a range of different ionic media, including chloroaluminate melts,^3^ imidazolium ionic liquids (ILs),^4^ and deep eutectic solvents (DESs),^5–11^ including with the presence of molecular additives or water. ILs are formed of salts with bulky organic cations that are liquid below 100 °C,^12^ whereas DESs are solvents formed from eutectic mixtures of quaternary ammonium salts and hydrogen bond donors.^13^ DESs have the same beneficial properties as ILs, such as good (electro)­chemical stability, low volatility, and high solubility of metal salts, that are critical to efficient electroplating electrolytes, but have the advantages of being formed from readily available components and are simpler to synthesize.^12^ These solvents have proved popular in the electroplating process due to their ability to deposit reactive metals and alloys, which are otherwise unattainable from aqueous systems.^12,14^ Additionally, their unique coordinating properties can alter the way metals nucleate and grow on surfaces. For example, electrodeposition from solutions of nickel chloride in a DES formed from choline chloride and ethylene glycol in a 1:2 molar ratio (ChCl/2EG) resulted in the formation of bright Ni coatings with fine grain size and mirror finish, having hardness values up to 460 HV.^5,6^ This is significantly higher than coatings from aqueous Watts nickel solutions (<350 HV). However, a key necessity of these DES-based plating systems is that plating must be carried out at a minimum of 80 °C; otherwise, a combination of thermodynamic and kinetic factors prevents nucleation within the potential window of the solvent. One of these factors is related to an unfavorable nickel species in solution. It is hence essential to understand nickel speciation and its temperature dependence in these electrolytes.

In the DESs investigated so far, thermochromism of the nickel species was observed above 80 °C,^5,15^ where the original complex undergoes reversible ligand exchange to form a chloride complex at around 120 °C. Similar behavior is also observed for nickel species in chloridic aqueous media^16^ and also in some ILs where there is a high concentration of non-chloride anions.^17^ This pattern of thermochromic behavior for nickel complexes is not limited to the chloride species, as shown by Lan et al. for mixed-ligand nickel-based ionic liquids,^18^ or when the nickel complex is embedded in a polymer.^19,20^

However, these previous studies do not attempt to identify the species present at the intermediate temperatures between room temperature and 120 °C and how these species affect the electrochemical behavior of the solution. In this work, we aim to answer the following questions.

- Are single mixed-ligand complexes present at intermediate temperatures, or is there a varying ratio of [Ni­(EG)_3_]^2+^ and [NiCl_4_]^2–^ complexes?
- In which temperature range do the nickel species switch from mainly octahedral to mainly tetrahedral coordination?
- How are the physical and kinetic parameters of nickel electrodeposition influenced by Ni ion speciation?

Extended X-ray absorption fine structure (EXAFS) is a technique that can elucidate the speciation of metal ions and has previously been used successfully in a range of ionic liquids and DESs.^21–23^ This method determines the average speciation in a sample, and in combination with the UV–vis data, we aim to identify the temperature at which the pure tetrachloride species is obtained and highlight where the main structural change from octahedral to tetrahedral coordination takes place.

## 2 Experimental Section

### 2.1 Solution Preparation

All DESs were made by stirring a 1:2 molar ratio of choline chloride (ChCl) (ABCR, 98%) with either ethylene glycol (EG) (Aldrich, ≥99%) or urea (U) (Grüssing, 99.5%) at 80 °C until a colorless homogeneous liquid was formed. These will be termed ChCl/2EG and ChCl/2U, respectively.

The solutions for extended X-ray absorption fine structure (EXAFS) measurements were made by stirring 0.1 mol dm^–3^ nickel chloride hexahydrate (Grüssing, 98%) with the relevant DES at 80 °C until the salt was fully dissolved. The samples used for UV–vis measurements were prepared to have a concentration of 0.01 mol dm^–3^ nickel chloride hexahydrate so that the absorbance was less than 4 units (saturation point of the detector), due to the intense coloration associated with tetrahedral complexes. The samples for electrochemical measurements used 0.05 mol dm^–3^ nickel chloride hexahydrate (Acros Organics, >98%) but were prepared in the same way.

### 2.2 Spectroscopic Methods

UV–vis spectra were measured on a JASCO V-670 spectrometer with SpectraManager software. Heated measurements were carried out with a Hellma fiber optic probe of 1 cm path length, with the sample held in an aluminum block to minimize fluctuations in temperature. Absorbance maxima were determined via Gaussian fitting in Origin 2015.

Extended X-ray absorption fine structure (EXAFS) spectroscopy was carried out by using the SpLine beamline (BM25A) at the ESRF synchrotron. Samples with a concentration of 100 mM were used to provide a good signal/noise ratio and ensure a clearly resolved edge-step in the EXAFS. The nominal K-edge energy for Ni was 8333 eV. Transmission data were measured with ionization chamber detectors and a double crystal Si(111) monochromator. To calibrate the amplitudes, the EXAFS spectrum of a Ni foil was recorded and fitted to known crystal structure data. Sample holders were made from two machined sections of PEEK fixed together with silicone glue, having a sample chamber size of 10 mm × 15 mm × 2 mm. The thickness of the PEEK “windows” was 0.2 mm. A heating foil with a thermal regulator was used to reach the working temperature. The Teflon and calcium silicate housing used as insulation were made in-house. The temperature was measured with a nichrome thermocouple immersed in the liquid sample, with an allowed temperature control with a maximum variation of ±1 °C over the course of each scan. No corrosion of the thermocouple was observed during the course of the measurements and can hence be considered to have negligible impact on the sample composition. Measurements were carried out at room temperature and at 10 °C intervals between 50 and 120 °C for the ChCl/2EG samples. Samples made with ChCl/2U could only be measured up to 110 °C, as thermal degradation of the liquid caused pressure buildup inside the cell, followed by sample leakage at higher temperatures. Three spectra were recorded for each sample, then averaged, calibrated, and background subtracted with Athena.^24^ The EXAFS spectra were fitted with Artemis to calculate the interatomic distances and their root-mean-square deviation (σ^2^). Electron scattering parameters were calculated to determine the type and number of coordinating atoms. Quoted uncertainties on fitted parameters are equal to two standard deviations.

The amplitude of the EXAFS signal is related to the number of nearby atoms multiplied by an amplitude dampening factor. This amplitude dampening factor was determined by fitting the measured EXAFS spectrum of nickel foil to the known crystal structure of nickel (ICSD Collection Code 53809).^25^ This value was calculated to be 0.8. The fitted k-range was from 2.7 Å^–1^ to ca. 10–12 Å^–1^, depending on the quality of the EXAFS signal.

### 2.3 Electrochemical Techniques

To determine current efficiency, cyclic voltammetry (CV) was carried out using scan rates from 5 to 50 mV s^–1^, in a temperature range of 20–120 °C. The working electrode was a 1 mm diameter platinum disc. Rotating disc electrode (RDE) experiments were carried out using an AUTOLAB RDE with platinum (3 mm), gold (3 mm), and glassy carbon (5 mm) disc electrodes surrounded by a Teflon sheath. To prevent any shear effects from the sides of the sample cell or effects from depletion of the analyte, all experiments were carried out using 0.5 L of solution. Due to the viscosity of the solvent, rotation rates were carefully selected to avoid the possibility of cavitation taking place. The rotation rate was therefore varied between 200 and 3000 rpm, using a potential scan rate of 10 mV s^–1^. The electrochemical measurements were carried out at 80 and 120 °C.

For both CV and RDE experiments, the counter electrode was a platinum flag, and the reference electrode was 0.1 mol dm^–3^ AgCl/Ag in ChCl/2EG, specifically designed for use in this DES. This reference electrode was separated from the solution to be analyzed with a glass frit. Potentials were then referenced to the [Fe­(CN)_6_]^3–/4–^ redox couple as an internal standard to permit comparison of the data to other systems.^26^ Prior to each experiment, the electrodes were polished with 0.3 μm γ-alumina paste, washed with deionized water, rinsed with acetone, and dried with air. The solutions were replenished upon each temperature investigation. All electrochemical measurements were carried out using a μAUTOLABIII/FRA2 impedance analyzer controlled using GPES software.

## 3 Results and Discussion

### 3.1 UV–Vis Spectroscopy

Temperature-dependent UV–vis data for solutions of nickel chloride hexahydrate in ChCl/2EG have been previously reported^5^ and are reproduced here in Figure 1a. At room temperature, the nickel species present is known to be [Ni­(EG)_3_]^2+^,^21^ whereas at high temperatures, the nickel ions are predicted to form tetracholoro-complexes. This hypothesis is supported by UV–vis spectroscopy, as the spectrum of nickel chloride in ChCl/2EG at 120 °C closely resembles that of nickel chloride in 1-hexyl-3-methylimidazolium chloride, [C_6_mim]­[Cl], at room temperature (Figure 1b), and in pyridinium chloride at 160 °C,^27^ both of which are known to form [NiCl_4_]^2–^ complexes under these conditions. The absorbance maxima present at 648 and 703 nm in ChCl/2EG and at 657 and 705 nm in [C_6_mim]­[Cl] are proposed to be related to the ^3^T_1_(F) → ^3^T_1_(P) transition, with the multiple absorbance maxima being due to spin–orbit splitting of the ^3^P state that arises from a distorted tetrahedral structure.^27,28^

**Figure 1:**
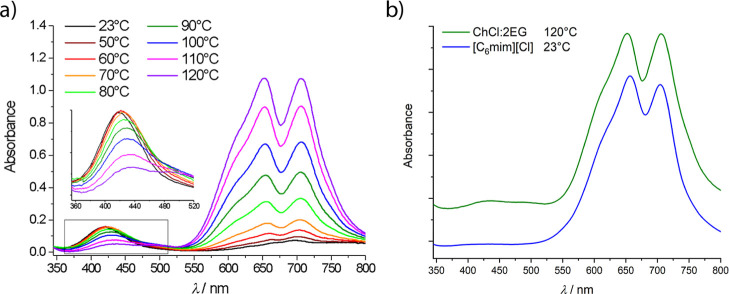
UV–vis spectra for solutions of 0.01 mol dm^–3^ NiCl_2_·6H_2_O in (a) ChCl/2EG at varying temperatures and (b) 120 °C ChCl/2EG and 23 °C [C_6_mim]­[Cl] (spectra offset for clarity). The peak wavelengths can be found in Table S1 in the Supporting Information.

As the temperature of the solution of nickel chloride in ChCl/2EG is increased, a color change from apple green to royal blue is observed, with a corresponding decrease in intensity for the absorbance between 400 and 500 nm, coupled with a shift to longer wavelengths (from 421 nm at 23 °C, to 470 nm at 120 °C). This observation is in agreement with the literature behavior of [C_4_mim]_2_[NiCl_4_] in [C_3_OHmim]­[BF_4_] or [C_4_mim]­[BF_4_], despite the initial higher proportion of chloro-complex at 25 °C in the literature data.^17^ This absorbance is most likely related to the ^3^A_2g_ → ^3^T_1g_(P) transition of the octahedral complex.

The absorption bands between 550 and 700 nm relating to ^3^A_2g_ → ^3^T_2g_(P),^3^E_g_(D) transitions^28^ increase in intensity with increasing temperature, with the maxima retaining similar wavelengths above 60 °C. A change from octahedral to tetrahedral is expected with the temperature increase, with the Laporte-permitted ^3^T_1_(F) → ^3^T_1_(P) transition dominating the spectrum. There is no clear isosbestic point between the two endpoint spectra of 23 and 120 °C, which suggests a stepwise ligand exchange instead of a mixture of the two “pure” complexes. Therefore, it is feasible that several species with different O-donor: chloride ratios are present at intermediate temperatures.

The corresponding ChCl/2U samples were cloudy due to gas bubbles formed from thermal decomposition of the urea at temperatures above 80 °C not dispersing due to the high viscosity of the liquid, resulting in spectra with poor signal-to-noise ratios. However, it was observed that the thermochromic change was not reversible for this solvent; instead, the solution remained a pale blue color, possibly due to coordination of the nickel ions to solvent decomposition products such as ammonia. This irreversible change is shown in Figure 2.

**Figure 2:**
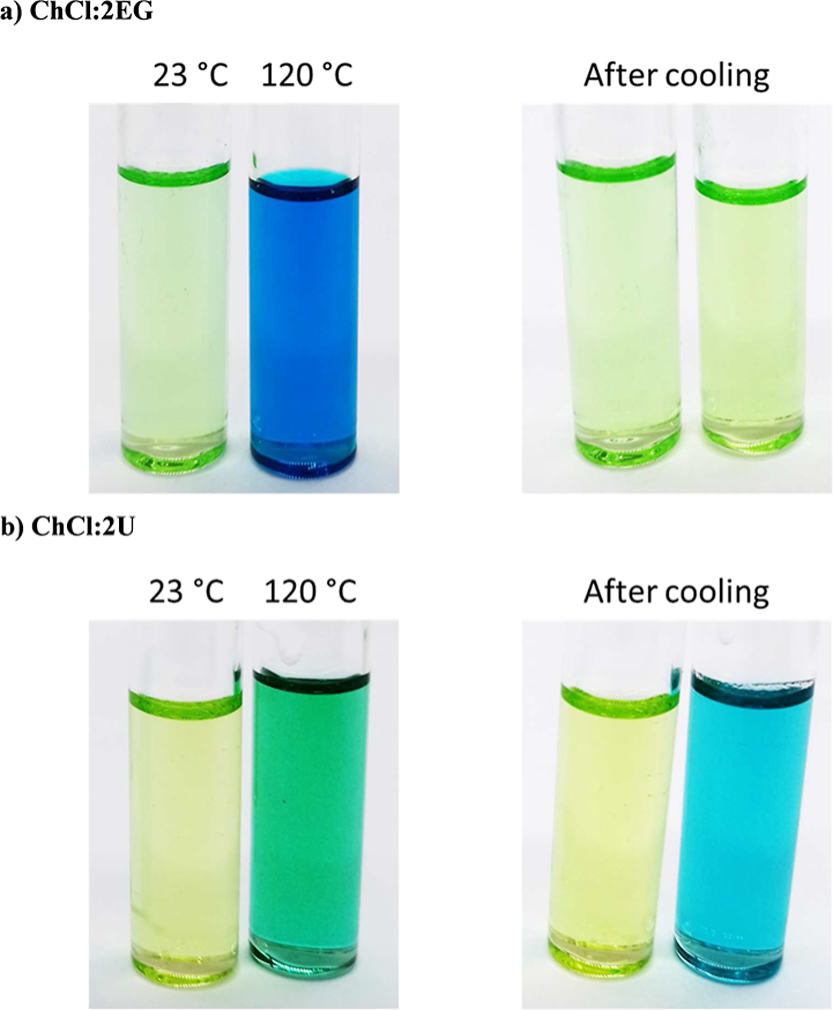
Photographs of solutions of 0.1 mol dm^–3^ NiCl_2_·6H_2_O in (a) ChCl/2EG and (b) ChCl/2U.

### 3.2 EXAFS Analysis of Nickel Chloride in ChCl/2EG

From previous EXAFS experiments, it is known that nickel ions chelate to EG-ligands in ChCl/2EG at room temperature (23 °C),^21^ and UV–vis data indicates that the thermochromic change from the EG complex to the tetrachloro complex should be complete by 120 °C. This hypothesis was confirmed, as the EXAFS spectrum for the solution of 0.1 mol dm^–3^ NiCl_2_·6H_2_O in ChCl/2EG at 120 °C could be fitted to 3.9(3) Cl-atoms at path lengths of 2.260(7) Å. These path lengths are comparable to the nickel tetrachloro-complexes present in imidazolium chloride systems (Ni–Cl path of 2.254(2) Å in [C_2_mim]­[Cl] or 2.272(6) Å in [C_6_mim]­[Cl]).^21,29^ No oxygen coordination was observed.

To determine the nickel species present in ChCl/2EG at the intermediate temperatures, a series fit was made using a model where the 23 °C system had a fixed coordination of six Ni–OC scattering paths, and the 120 °C system had a fixed coordination of four Ni–Cl scattering paths. Due to the correlation between the coordination number (CN) and σ^2^ parameters within the fitting program, only one σ^2^ value was permitted per atom type. As σ^2^ is a measure of thermal disorder, it would usually be expected to increase with an increasing temperature. However, permitting variation for each individual scattering path at each temperature (an additional 18 parameters) resulted in a poor fit. The first Ni–O–C multiple scattering path was also included in the fitting model, as chelation of the nickel ions by EG is expected at lower temperatures. This shows as a shoulder at ca. 2.8 to 3.0 Å on the main peak in the Fourier transform.^21^ However, no O–O, O–Cl, or Cl–Cl scattering paths were included, as these would require prior knowledge of the geometry of each species involved and would be expected to have only a weak contribution to the overall spectrum.

Coordination number and Ni-ligand path lengths were refined for each intermediate temperature, with the results shown in Table 1. Linear combination fits of the near-edge region (Figures S1a) support these results and are shown graphically as Figure 3. The graphs for the individual linear combination fits can be found in Figure S2 of the Supporting Information Spectra for selected temperatures are shown in Figure 4. The spectra and fits of the full temperature data series, including the model scattering paths used during the fitting process, can be found in Figure S3 of the Supporting Information. Small amounts of chloride appear immediately in the nickel complex with an increase in temperature, with an O/Cl ratio of approximately 5:1 present up to 70 °C. Above 80 °C, the O/Cl ratio in the nickel complex indicates that multiple species are likely to be present, and this behavior persists until 120 °C. The critical temperature for a major change in nickel speciation appears to be between 90 and 100 °C, as the complex geometry changes from mostly six-coordinate to mostly four-coordinate. The majority species most likely to be present are proposed in Table 1. Where atom fractions are present in the coordination number, this indicates that a mixture of species is more likely to be present than not. However, as EXAFS is an average technique, we do not attempt to determine the exact ratios of the species here. The detailed mechanistic steps involved in the conversion from the Ni glycolate complex to the tetrachloride complex are not known here but may involve nucleophilic attack by free chloride ion, since this is generally assumed to be a stronger nucleophile. This would form the basis of a separate study. These findings correlate well with our subsequent findings and also with previous electrochemical analysis, which both show that a temperature of 80–90 °C is required for the facile electrodeposition of compact, adherent, and bright nickel coatings from solutions of ChCl/2EG.^5,6^

**Table 1:** EXAFS Fit Parameters for Solutions of 0.1 mol dm^–3^ NiCl_2_·6H_2_O in ChCl/2EG at Different Temperatures, Assuming the Presence of Cl^–^ in all Heated Samples^b^

Temp, °C	Coordinating atom/group	Number of atoms, CN	Distance from Ni, *r*/Å	Proposed species
23	OC	6^a^	2.087(7), 2.89(2)	[Ni(EG)_3_]^2+^≡[Ni(OD)_6_]^2+^
50	OC	5.0(4)	2.07(1), 2.88(3)	[Ni(OD)_5_Cl]^+^
	Cl	1.2(4)	2.35(2)	
70	OC	4.7(5)	2.07(2), 2.87(4)	[Ni(OD)_5_Cl]^+^
	Cl	1.2(5)	2.34(2)	
80	OC	4.2(5)	2.07(2), 2.87(4)	[Ni(OD)_4_Cl_2_]
	Cl	1.5(5)	2.32(2)	& [Ni(OD)_5_Cl]^+^
90	OC	3.5(6)	2.06(2), 2.85(5)	[Ni(OD)_4_Cl_2_]
	Cl	1.9(5)	2.30(2)	& [Ni(OD)_3_Cl_2_]
100	OC	2.9(7)	2.06(2), 2.82(6)	[Ni(OD)_3_Cl_2_]
	Cl	2.2(5)	2.29(1)	
110	OC	2.2(1.0)	2.07(3), 2.78(9)	[Ni(OD)_2_Cl_3_]^−^ & [Ni(OD)Cl_3_]^−^
	Cl	2.6(7)	2.27(2)	
120	Cl	4^a^	2.260(7)	[NiCl_4_]^2–^

a Coordination numbers are fixed to 6 and 4 for the “pure” OC and Cl complexes, respectively. σ2 refined to O = 0.0046(5), C = 0.009(2), and Cl = 0.0058(5). *R*-factor = 0.0084 and reduced chi2 = 67.

b Note that EG here refers to ethylene glycol acting as a bidentate ligand, and OD refers to it acting as a monodentate ligand.

**Figure 3:**
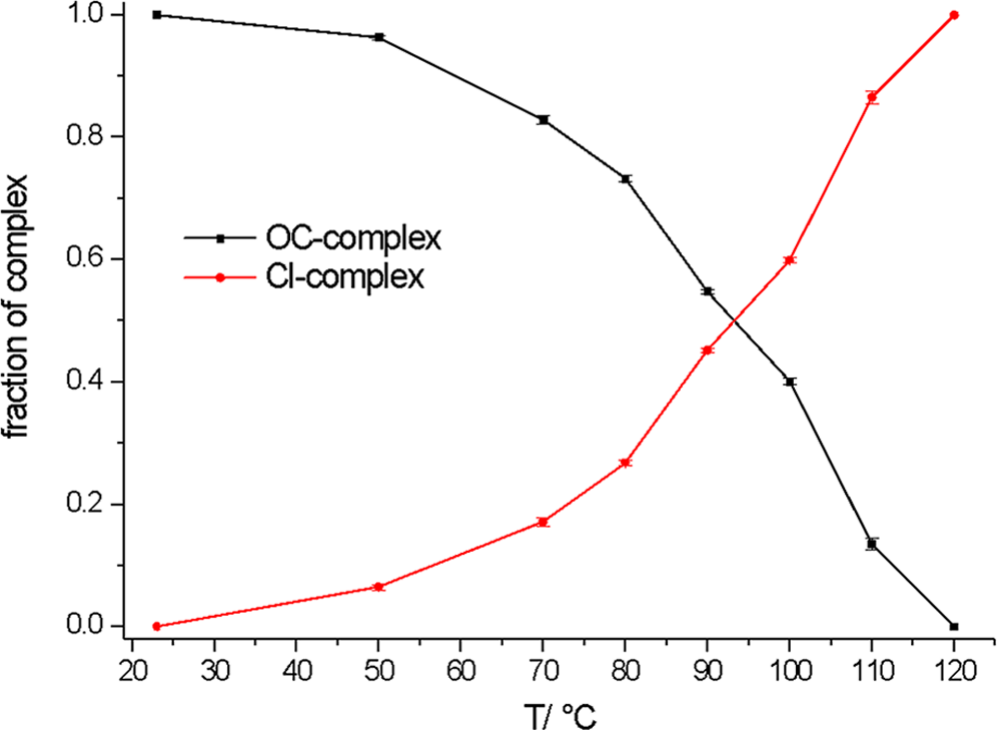
Fraction of coordinated O- and Cl-atoms in the nickel complexes in ChCl/2EG as a function of temperature, obtained from linear combination fitting of the near edge region shown in Figure S1a.

**Figure 4:**
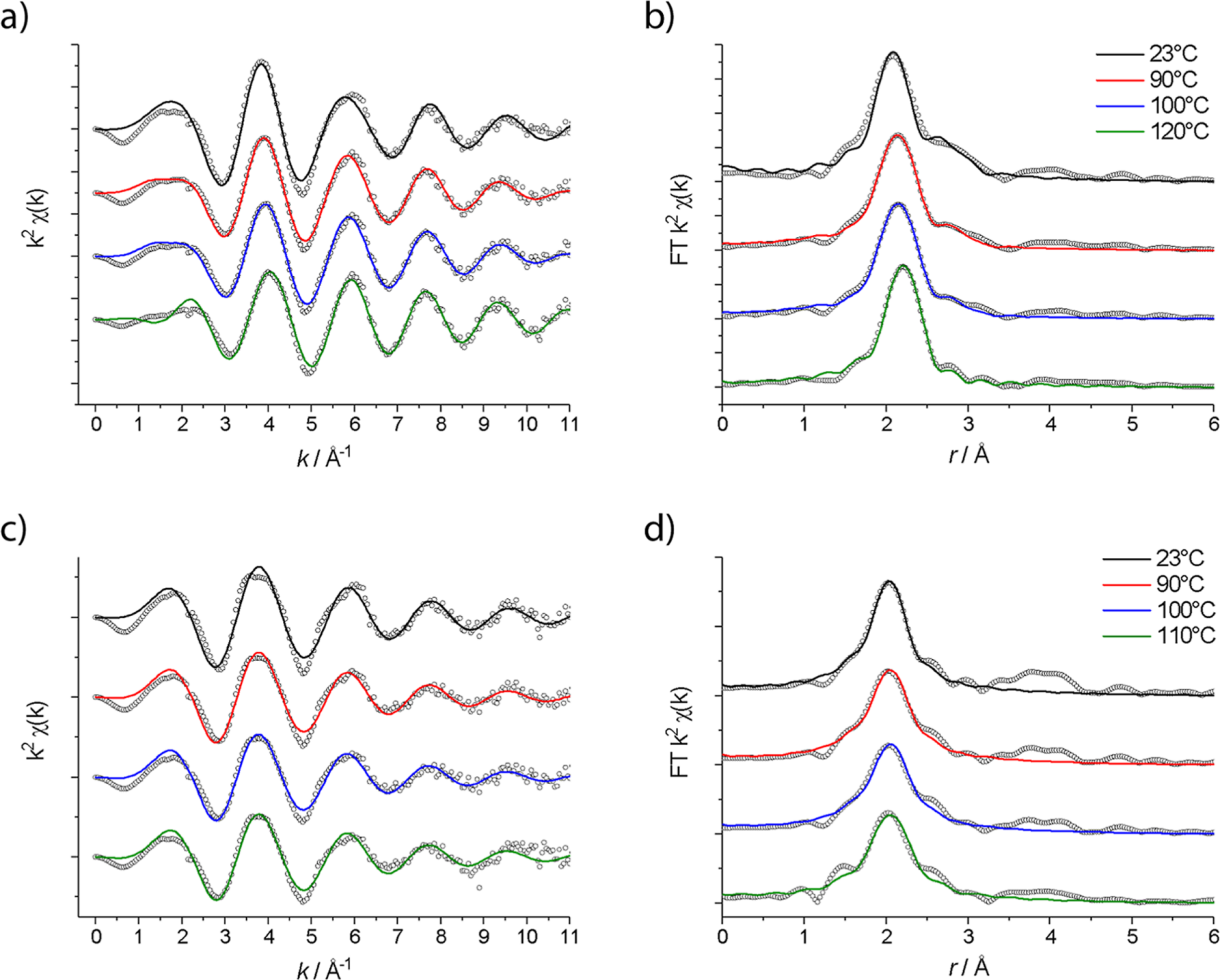
*k*2-weighted EXAFS (a,c) and Fourier transform (b,d) of 0.1 mol dm^–3^ NiCl_2_·6H_2_O in ChCl/2EG (a,b) and ChCl/2U (c,d) at selected temperatures. Data are dots; fits are lines. Spectra are offset for clarity.

Counterintuitively, the Ni-ligand path lengths appear to decrease with temperature in these systems. Higher temperatures generally result in longer, more disordered metal–ligand paths. However, in the examples investigated here, the apparent contraction in Ni-ligand path length is more likely to be related to the geometric change from a bulky six-coordinate complex with a radius of at least 2.9 Å to a four-coordinate complex with a radius of at least 2.26 Å.

### 3.3 EXAFS Analysis of Nickel Chloride in ChCl/2U

Irreversible thermochromic changes spanning a color change from green to pale blue were observed for nickel salts in ChCl/2U at temperatures above 100 °C. Variation of the spectrum in the near-edge region with temperature supports the nickel ion slowly changing speciation with increasing temperature (Figure S1b). However, comparison of the spectrum of 0.1 mol dm^–3^ NiCl_2_·6H_2_O in ChCl/2U at 110 °C to that of the corresponding ChCl/2EG system shows little similarity, indicating that the new high-temperature complex in the urea DES is unlikely to be a tetrachloro-complex.

All of the spectra for 0.1 mol dm^–3^ NiCl_2_·6H_2_O in ChCl/2U display a single peak at ca. 2.08 Å, which can be fitted to 5 to 6 ligands with N- or O-coordinating moieties, regardless of temperature (Figure 4). The full data series can be found in Figure S4 of the Supporting Information, including the model scattering paths used during the fitting process. Nitrogen and oxygen have very similar electron densities; hence, it is not possible to distinguish between them with EXAFS. Therefore, it cannot be determined from these data whether the nickel ions are coordinated to the carbonyl or amine moieties of the urea. Scattering path lengths and σ^2^ values both increase with an increase in temperature, as expected for a system with higher disorder. No Cl^–^-coordination was observed at any temperature. The data fits are suggestive of a decrease in CN with the temperature (Table 2). However, this decrease in fitted CN values could be due to a physical change in coordination number or an artifact arising from the inherently high correlation between CN and σ^2^, resulting in an artificial change or the exchange of the ligands present at low temperatures for lighter coordinating atoms.

**Table 2:** EXAFS Fit Parameters for Solutions of 0.1 mol dm^–3^ NiCl_2_·6H_2_O in ChCl/2U, with Varying Temperature^a^

Temp, °C	Coordinating atom/group	Number of atoms, CN	Distance from Ni, *r*/Å	Debye–Waller factor, σ^2^/Å^2^
23	O/N	5.5(4)	2.07(1)	0.006(1)
50	O/N	5.6(4)	2.08(1)	0.007(2)
70	O/N	5.5(4)	2.079(9)	0.007(1)
80	O/N	5.4(4)	2.08(1)	0.008(2)
90	O/N	5.2(4)	2.08(1)	0.008(2)
100	O/N	5.0(5)	2.09(1)	0.008(2)
110	O/N	4.9(5)	2.09(1)	0.008(2)

a *R*-factor = 0.0121 and reduced chi^2^ = 51.

Unlike the spectra for the ChCl/2EG systems, no shoulder is observed on the main peak (Figure 4d), i.e., no chelating ligands. Instead, a broad set of signals is observed at ca. 4 Å, which can be assigned to the presence of urea ligands. Similar behavior was also observed for samples of a CrCl_3_·6H_2_O: urea eutectic liquid^30^ and for manganese chloride in ChCl/2U.^21^ The intensity of this signal decreases with the temperature, indicating the presence of a new species with less urea coordination. As urea thermally decomposes to ammonia, it is likely that the pale blue color is due to the formation of a nickel ammine complex. These signals have not been modeled due to the unknown geometry of urea coordination to the Ni^2+^ ion and the complexity of the resulting multiple scattering paths.

This difference in thermochromic reversibility for nickel ions in ChCl/2U and ChCl/2EG can be explained by considering the stability constants of the different species formed. While these data are not currently available for DES media, aqueous systems can be used as a qualitative guide. In room temperature aqueous media, the stability of the nickel hexaammine complex is high (log β = 9.33)^31^ in comparison to the nickel tetrachloro-complex (log β = −3.05 to −3.38).^16,32^ Therefore, once the effect of heating is removed, the tetrachloro-complex is so thermodynamically unfavorable that the nickel ethylene glycol complex reforms, whereas the nickel hexaammine complex remains.

### 3.4 Effect of Speciation on Electrochemical Properties

It has previously been concluded that the rate of nickel electrodeposition in DES media is controlled by kinetic factors, such as the formation of a passivating oxide layer, rather than by thermodynamic factors.^11,33^ However, metal speciation can also play a role through both the reactivity of the species and also the charge on the species. Critically, the EXAFS data fits have shown that the nickel species in ChCl/2EG change from cationic to anionic above 100 °C, which will alter the interactions between the nickel complex and the double layer formed at the electrode surface. For example, at cathodic potentials in ChCl/2EG, a layer of choline cations is known to form at the electrode surface, with charged groups orienting themselves closer to the electrode and with the alcohol groups closer to the bulk solvent.^34^ This will result in the electrode surface being made poorly accessible for the bulkier cationic octahedral complex compared to the smaller anionic tetrahedral complex. Hence, the electrodeposition should become more favorable when greater amounts of the tetrachloride species are present.

Cyclic voltammograms of the nickel ChCl/2EG solutions at different temperatures are presented in Figure 5. The CVs show generic features, including a cathodic reduction wave at ca. −0.5 V corresponding to the deposition of Ni metal coating on the electrode and an anodic wave at ca. +0.2 V for the corresponding dissolution of the deposited Ni. The CVs clearly show that at higher temperatures, both sets of waves are both larger in magnitude and more clearly defined. This is consistent with previous observation^5^ and is partly due to expected trends in mass transport and viscosity. The CVs also show that the onset potential for the reduction wave becomes more anodic with increasing temperature, i.e., decreasing the required electrodeposition overpotential, by up to ca. 200 mV difference between the 20 and 120 °C voltammograms.

**Figure 5:**
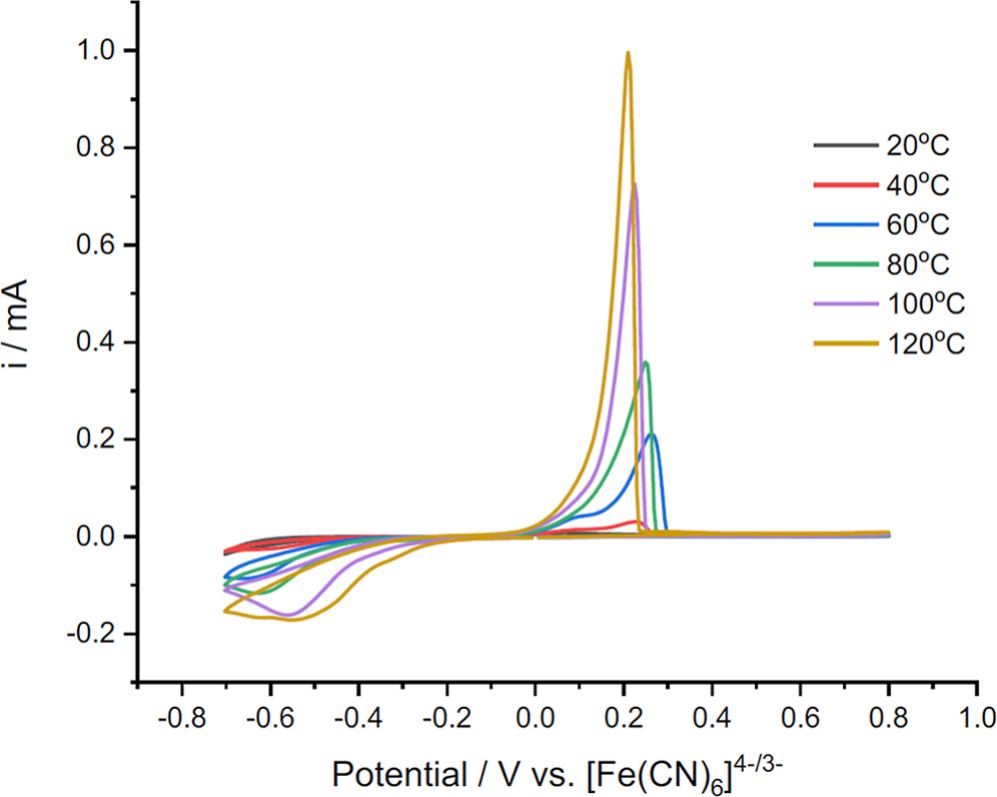
Cyclic voltammograms of solutions of 0.05 mol dm^–3^ NiCl_2_·6H_2_O in ChCl/2EG at temperatures between 20 and 120 °C on a 1 mm Pt-disc working electrode, referenced against the [Fe­(CN)_6_]^3–/4–^ couple as an internal standard. The counter electrode was a Pt flag, and the scan rate was 10 mV s^–1^.

Temperature increases are known to affect measured redox potentials, as described by the Nernst eq (eq 1)­

1 $$E=E^{0}+\frac{RT}{nF}\ln\left(\frac{a_{ox}}{a_{red}}\right)$$

where *E* is the measured potential, *E* ^0^ is the formal electrode potential, *R* is the gas constant, *T* is temperature, *n* is the number of electrons, *F* is the Faraday constant, *a*_ox_ is the activity of the oxidized species, and *a*_red_ is the activity of the reduced species. To determine whether the increased temperature was responsible for the observed anodic shift of 200 mV, predictions for the expected deviations were calculated. At a solution concentration of 0.05 mol dm^–3^ Ni^2+^ species and a temperature of 25 °C, this is equivalent to a shift of −0.04 V. At a temperature of 120 °C, this is equivalent to a shift of −0.05 V. This is only a variation of 10 mV. Therefore, contributions from temperature alone cannot be responsible for the large reduction in onset potential shift observed here.

On the anodic dissolution sweep, two features are visible in the CVs at the lower temperature solutions (from 20 to 60 °C). These can be associated with the stripping of two morphologies of nickel deposits formed due to edge effects, where higher currents are concentrated at the edge of the working electrode. This has been proposed to be related to the hemispherical diffusion of solution species.^35^ In contrast, above 100 °C, a single sharp anodic stripping peak is observed. A contributory factor is the improved diffusion of nickel solution species from the decreased solvent viscosity. In addition, and importantly here, this change in behavior also occurs over the temperature range (90 and 100 °C) during which the nickel coordination changes from mainly octahedral to mainly tetrahedral species.

Viscosity values have previously been determined for solutions of ChCl/2EG containing NiCl_2_·6H_2_O between the temperatures of 25 and 85 °C, along with calculated activation energies.^36^ These viscosity values decrease from ca. 135 mPa s at 25 °C to ca. 11 mPa s at 85 °C. Diffusion is related to viscosity by the Stokes–Einstein eq (eq 2)­

2 $$D=\frac{k_{B}T}{6\pi \eta r}$$

where *D* is the diffusion coefficient, *k*_B_ is the Boltzmann constant, η is viscosity, and *r* is the Stokes radius of the spherical particle. It can be seen that the diffusion coefficient is proportional to the inverse of viscosity; therefore, if viscosity decreases by a factor of 10, the diffusion coefficient is expected to increase by an order of magnitude.

However, according to the Cottrell eq (eq 3), the diffusion coefficient of a solution species is also proportional to the square of the current

3 $$i=\frac{nFAc^{0}\sqrt{D}}{\sqrt{\pi t}}$$

where *i* is current, *A* is electrode area, *c* ^0^ is initial concentration of the reducible analyte, and *t* is time. Hence, for a change in diffusion coefficient by an order of magnitude, it would be predicted that current should increase by three to four times. The anodic current passed at 120 °C in Figure 5 is approximately 20 times larger than that at 30 °C. This increase in current is significantly larger than would be expected if physical properties of the solution were responsible for the increase in current.

Ultimately, while the improved electrochemical response observed for nickel electrodeposition from ChCl/2EG at increased temperatures may contain contributory responses from the decreased viscosity and improved mass transport, the change in reduction onset potential and amount of nickel deposited at 120 °C compared to those at 25 °C are significantly larger than predicted. Therefore, this electrochemical response must be due to changes in coordination.

The current efficiency, or Coulombic yield, of an electrochemical reduction/dissolution process is a useful metric of performance. In protic solvents (water or DES) where metal-ion reduction kinetics are slow or rate-limiting, it can often be observed that current efficiencies are quite low. For example, in the commercial electroplating of Ni and Cr metals, the current efficiency can be as low as 40%, where the remainder of the Faradaic charge is consumed by side reactions such as proton reduction (and hydrogen evolution), which are relatively facile. Here, current efficiency was calculated by numerical integration of the cathodic and anodic charges associated with the voltammogram, eq 4, where Ni_Ox_^0^ is the integral of the oxidative process and Ni_Red_^2+^ is the integral of the cathodic process

4 $$current\;efficiency\;\left(\%\right)=\frac{{Ni}_{Ox}^{0}}{{Ni}_{Red}^{2+}}\times 100$$

Current efficiency data for the Ni ion solution in ChCl/2EG DES are presented as Figure 6 as a function of temperature at a fixed experimental time scale (i.e., potential scan rate). The data in Figure 6 clearly show a strong dependence of the current efficiency on temperature. A current efficiency of 80–90% is determined at high temperature, and this correlates well with previous observations^5^ and with the trend in the CVs, Figure 5. Over the relatively low temperature range (40–60 °C) this might be due to improved mass transport and associated thermal activation trends. However, if these were the only determining factors, then we would anticipate that the trend would be continuous and monotonic. However, Figure 6 shows that while current efficiency increases between 40 and 60 °C and between 80 and 100 °C, there are efficiency “plateaus” at 60–80 °C, and at 100–120 °C. These temperature ranges are where distinct species types are present, i.e., below 60 °C [Ni­(EG)_3_]^2+^ is present; between 60 and 80 °C, there is a mixture of [Ni­(OD)_4_Cl_2_] and [Ni­(OD)_5_Cl]^+^, whereas above 100 °C tetrahedral species dominate. This strongly suggests that the nickel species plays an important role in the efficiency of nickel electrodeposition in ChCl/2EG.

**Figure 6:**
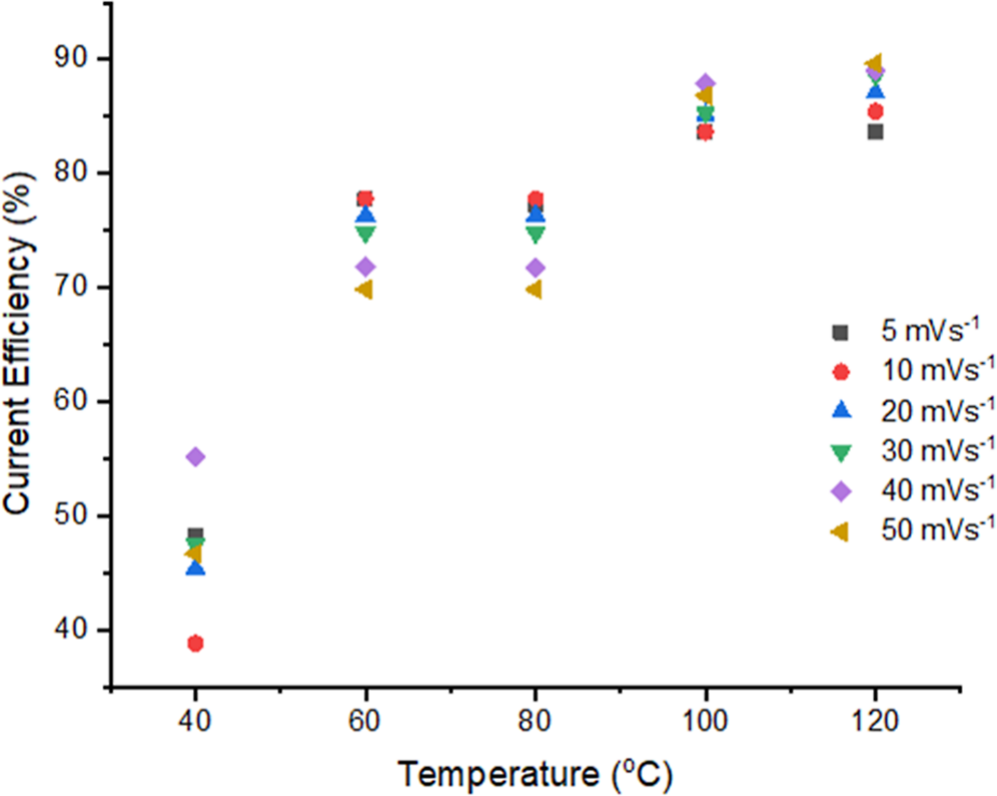
Static measurement current efficiency plots for 0.05 mol dm^–3^ NiCl_2_·6H_2_O in ChCl/2EG as a function of temperature. CVs recorded on a 1 mm Pt-disc working electrode, referenced against the [Fe­(CN)_6_]^3–/4–^ couple as an internal standard, with a Pt flag counter electrode.

In an attempt to better understand the kinetics of nickel electrodeposition from ChCl/2EG, rotating disc electrode (RDE) voltammetry was carried out at selected temperatures of 80 °C (the highest temperature at which the octahedral complex still dominates) and 120 °C (the temperature where only the tetrahedral complex is present). RDE voltammetry is a hydrodynamic technique where a laminar flow is generated by rotating the working electrode. A steady-state system is achieved where mass transport is controlled by solution flow rather than diffusion of the conducting species. Under such conditions, the diffusion coefficient of the electrochemically active species in solution, *D*_0_, can be calculated using the Levich eq (eq 5), which describes the relationship between measured current *i*_L_ and electrode angular velocity ω of rotation

5 $$i_{L}=0.62nFAC_{0}{D_{0}}^{2/3}v^{-1/6}\omega^{-1/2}$$

where *n* is the number of electrons, *F* is the Faraday constant, *A* is the electrode area, *C*_0_ is the analyte concentration, *v* is the kinematic viscosity of the solvent, and ω is the angular rotation rate of the electrode.

RDE voltammetry data for Ni ion solution in the ChCl/2EG system as a function of rotation rate are presented in Figure 7a (at 80 °C) and Figure 7b (at 120 °C). In a model system, where electron-transfer kinetics are facile and hence not rate-limiting, the RDE curve should adopt a sigmoidal S-shape where the current response at the most cathodic potentials is independent of potential, i.e., controlled by mass transport only. In such a model system, this limiting condition is often approached only at high rotation rates. It is clear from the data in Figure 7 that this condition is not reached in our system at either temperature. The current responses of both data sets (both temperatures) show significant curvature at the cathodic potentials over the full range of rotation rates. Unfortunately, this precludes further quantitative analysis using the usual methodologies (eq 5). However, qualitative interpretation of these data sets indicates that while both exhibit evidence of continued kinetic control, this is much more prominent at the lower temperature. This leads us to conclude that the kinetics of reduction for the 4-coordinate Ni tetrachloride species is faster and more favorable for metal ion reduction.

**Figure 7:**
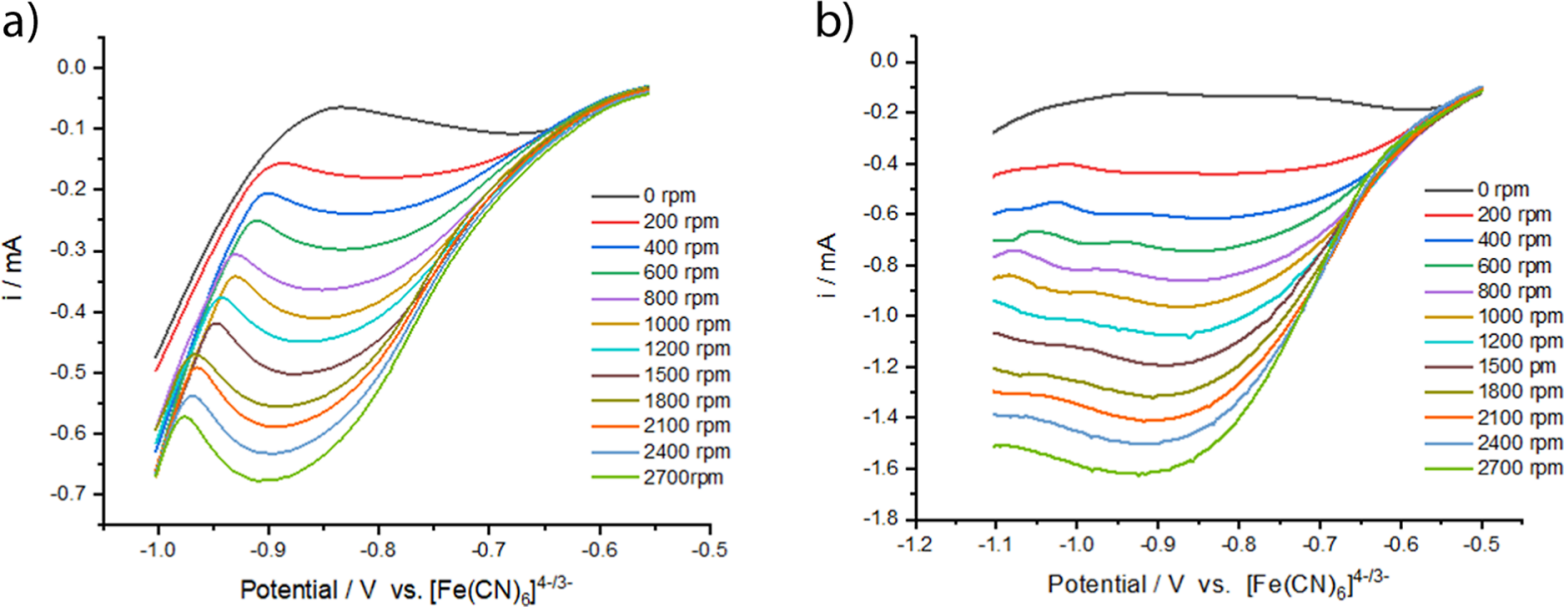
Rotating disc electrode voltammetry for 0.05 mol dm^–3^ NiCl_2_·6H_2_O in ChCl/2EG at (a) 80 °C and (b) 120 °C using a 3 mm diameter Pt disc working electrode, measured at a scan rate of 10 mV s^–1^. The counter electrode was a Pt flag, and scans are referenced against the [Fe­(CN)_6_]^3–/4–^ couple as an internal standard.

For the example of the 80 °C system, once the diffusion-limited regime is reached, the current begins to decrease, indicative of a kinetic hindrance to nickel deposition (Figure 7a). Common kinetic hindrance issues include the enrichment of the double layer with “free” ligands, passivation of the electrodeposit by solvent electrolysis products (e.g., hydrogen evolution from electrolysis of the glycol),^37^ or an effect of the substrate material. As the investigated systems approach high angular rotation speeds, a greater curvature in the current profile is observed, as would be reasonably expected by the larger amount of both the solvent and nickel complex reaching the electrode. At large overpotentials (ca. −0.85 to −1.0 V, depending on angular rotation), a sharp current increase is detected that can be linked to decomposition of the DES. This solvent decomposition process is not observed at 120 °C (Figure 7b), potentially due to the decreased viscosity of the solvent and smaller electrodeposition overpotential. The corresponding measurements at an Au working electrode are qualitatively similar and can be seen in Supporting Information Figure S5. Stationary voltammetry measurements of the solvent at 120 °C are shown in the Supporting Information Figure S6.

## 4 Conclusions

The thermochromic behavior of nickel chloride in two choline chloride-based DESs was investigated at a range of temperatures from 23 to 120 °C via UV–vis and EXAFS spectroscopies. In ChCl/2EG, it was observed that nickel showed reversible thermochromism, changing from [Ni­(EG)_3_]^2+^ at room temperature, to [NiCl_4_]^2–^ at 120 °C, most likely via a stepwise ligand exchange. The switching point between mainly octahedral geometry and mainly tetrahedral geometry was determined to be between 90 and 100 °C, corresponding to the ideal temperatures required for obtaining good nickel electrodeposits from these systems. In ChCl/2U, however, irreversible thermochromism took place due to thermal decomposition of the urea component of the DES and the formation of a blue nickel–ammonia complex.

Cyclic voltammetry shows that the current efficiency of nickel electrodeposition generally increases with temperature but also that the increasing fluidity and decreasing barriers to mass transport are not the main driving force. Instead, the effect of changing speciation can be seen as current efficiency is similar across the temperature range where similar species are present. RDE voltammetry was carried out initially to determine the diffusion coefficients for [Ni­(EG)_3_]^2+^ and [NiCl_4_]^2–^. However, a steady state could not be reached for either the 80 or 120 °C measurements due to the complexity of the multiple reduction reactions taking place. It should be noted that electrochemical reduction of the nickel species is enhanced relative to electrochemical decomposition of the solvent at higher temperatures. The insights gained in this study will be relevant to the application of DES in commercial electroplating challenges and may also be useful in optimizing existing aqueous baths.

## Supplementary Material



## Notes

Additional EXAFS fitting methodology, fitted EXAFS spectra, and rotating disc electrode plots (PDF)
