# Supplementary material for: EXAFS and Rotating Disc Electrode Study into the Thermochromic Behavior of Nickel Salts in Deep Eutectic Solvents
Source: J Phys Chem C Nanomater Interfaces. 2025 Nov 11;129(46):20758–67. doi: 10.1021/acs.jpcc.5c05771 (PMC12641486; doi:10.1021/acs.jpcc.5c05771)
Supplement: Supplementary file 1 [file jp5c05771_si_001.pdf]

EXAFS and Rotating Disc Electrode Study into the Thermochromic Behaviour of Nickel Salts in Deep Eutectic Solvents

Jennifer M. Hartley,[a, b*] George Tebbutt,[a] Andrew Ballantyne,[c] Charlotte Ashworth-Güth,[b] Gero Frisch,[b] Karl S. Ryder[a]

[a] School of Chemistry, University of Leicester, Leicester, LE1 7RH, UK

[b] Institut für Anorganische Chemie, TU Bergakademie Freiberg, 09599 Freiberg, Germany

[c] School of Geology, University of Leicester, Leicester LE1 7RH, UK

* Contact email: jmh84@le.ac.uk

Supplementary information

Table S1: Absorption maxima for nickel UV-vis spectra presented in **Figure 1**, fitted using Origin2015.

**ChCl:2EG**				
**Temperature**	**Peaks / nm**			
23	421	-	692	786
50	424	-	694	797
60	426	-	690	794
70	427	616	651	698
80	428	616	651	701
90	432	617	651	703
100	435	617	651	705
110	438	617	651	705
120	470	613	648	703
130	-	616	650	705
**[C_6mim][Cl]**				
**Temperature**	**Peaks / nm**			
23	-	-	657	705

Linear combination fitting of the XANES region

ChCl:2EG

Linear combination fitting (LCF) of the near edge region of the intermediate temperature spectra (**Figure S1a**) was carried out in the program Athena.[1] This involves comparing how much the individual spectra are similar to the "end members" of known 6-coordinate and known 4-coordinate spectra. It was observed that the white line was reduced in amplitude and shifted to smaller energy values as temperature was increased, whilst a pre-peak around 8330 eV increased in magnitude with increasing temperature. In addition, the intensity of the oscillations is reduced with temperature, characteristic of a switch from coordination by lighter to heavier atoms. This behaviour is very similar to that of hydrothermal nickel chloride systems, despite the different solvent, temperatures, and pressures used.[2] LCF indicated that a major structural change takes place between 90 and 100 °C.

ChCl:2U

Variation of the spectrum in the near edge region with temperature supports the nickel ion slowly changing speciation with increasing temperature, but the complex present remains roughly the same geometry (**Figure S1b**). The new high temperature complex is highly unlikely to be a tetrachloro-complex. Instead, it is more probable that the species is related to decomposition products of the urea, such as ammonia.

a) b)

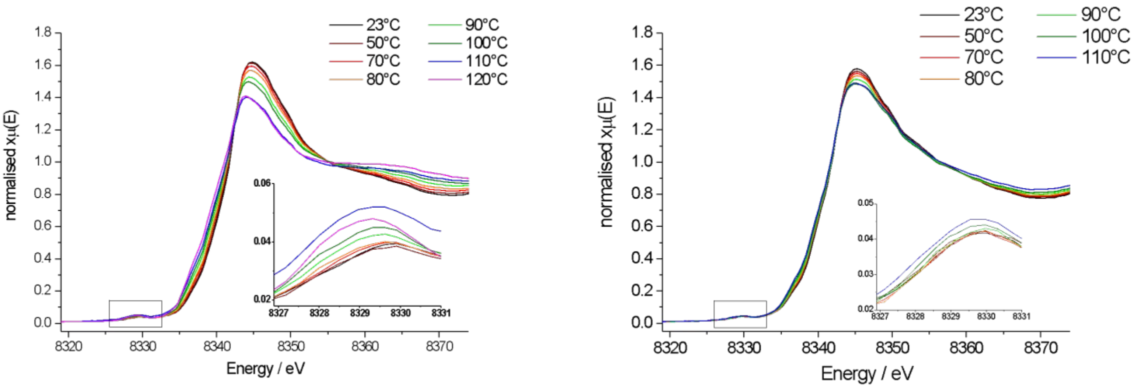

Figure S1: XANES region of solutions of 0.1 mol dm^{-3} $NiCl_2{\cdot}6H_2O$ at different temperatures in a) ChCl:2EG, and in b) ChCl:2U. Inset: Expanded pre-edge peak.

a) T = 50 °C

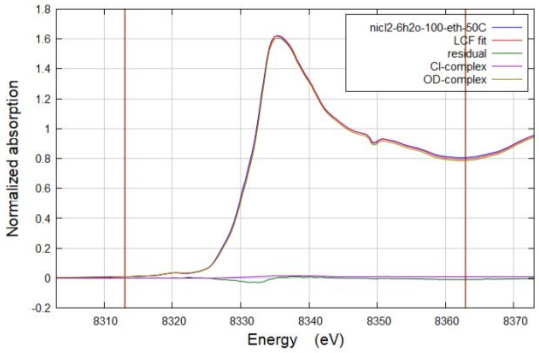

b) T = 70 °C

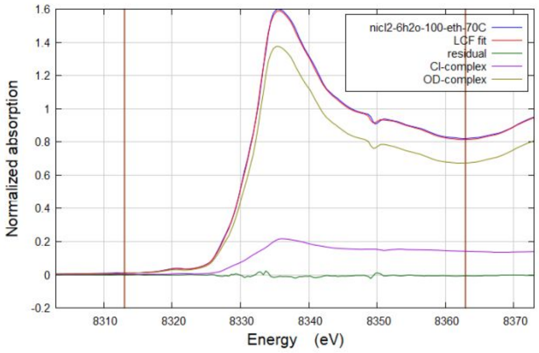

c) T = 80 °C

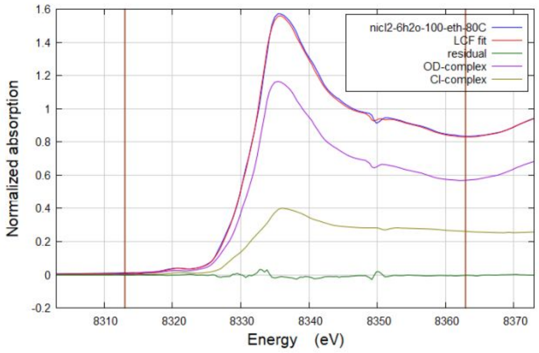

d) T = 90 °C

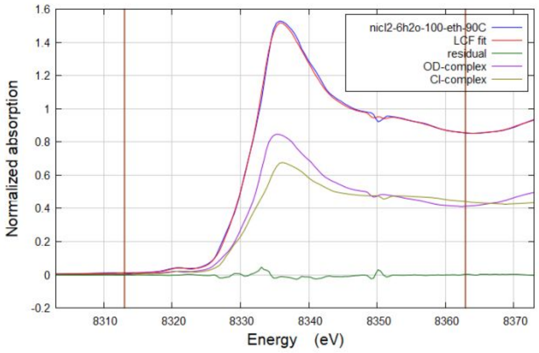

e) T = 100 °C

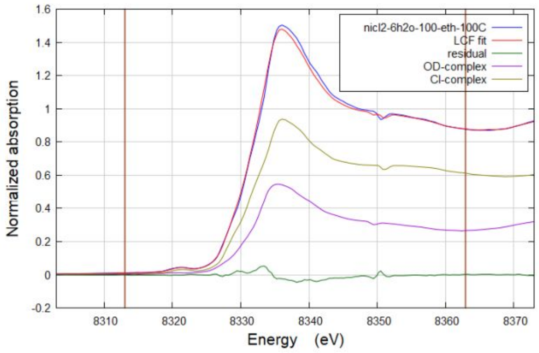

f) T = 110 °C

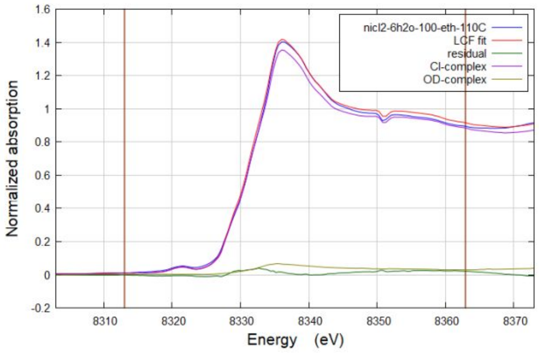

Figure S2: Linear combination fits of the XANES region of solutions of 0.1 mol dm^{-3} $NiCl_2 \cdot 6H_2O$ in ChCl:2EG, at temperatures between 50 and 110 °C, assuming that the species at 23 °C is 100% EG-coordinated and the species at 120 °C is 100% Cl-coordinated.

a) ChCl:2EG at 23 °C (k²-space)

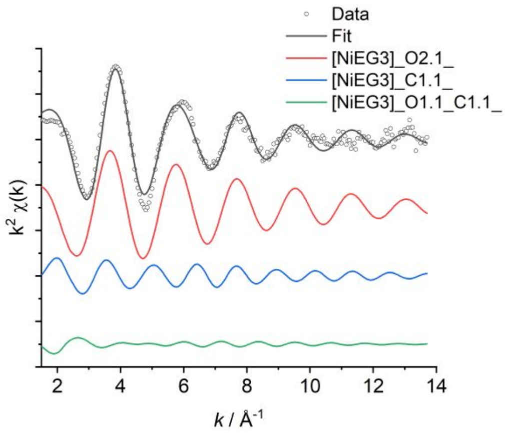

b) ChCl:2EG at 23 °C (r-space)

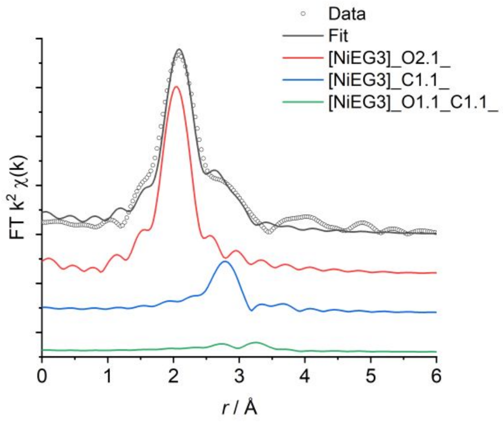

c) ChCl:2EG at 50 °C (k²-space)

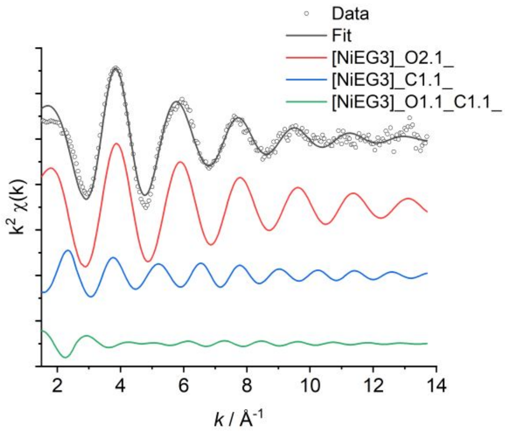

d) ChCl:2EG at 50 °C (r-space)

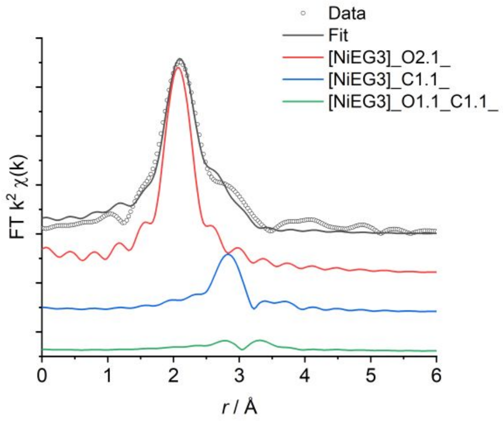

e) ChCl:2EG at 70 °C (k²-space)

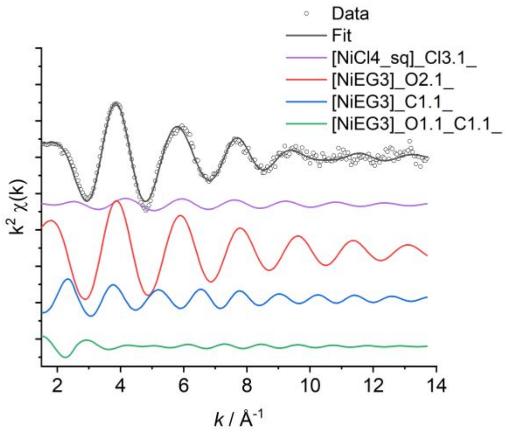

f) ChCl:2EG at 70 °C (k²-space)

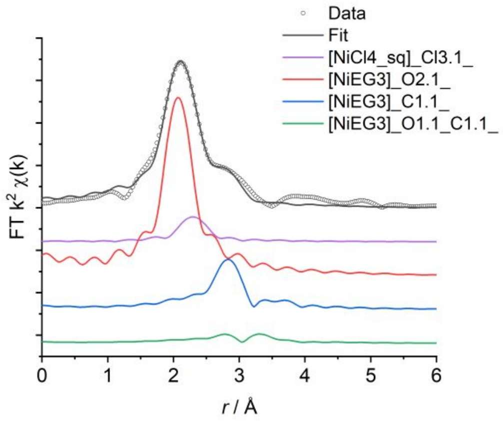

Figure S3: k^2-weighted EXAFS (left) and Fourier transform (right) of 0.1 mol dm^{-3} $NiCl_2{\cdot}6H_2O$ in ChCl:2EG, at temperatures between 23 and 120 °C. Data are dots, fits are lines. Model scattering paths used during fitting offset for clarity.

g) ChCl:2EG at 80 °C (k²-space)

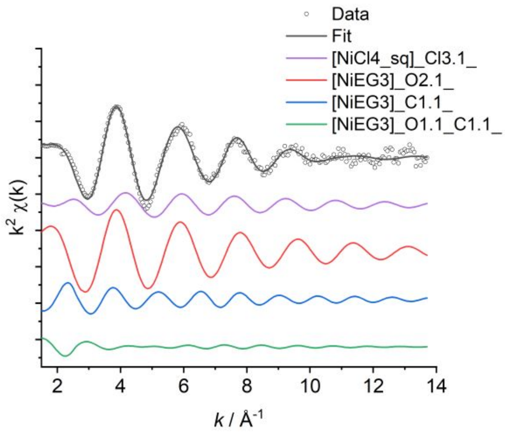

h) ChCl:2EG at 80 °C (r-space)

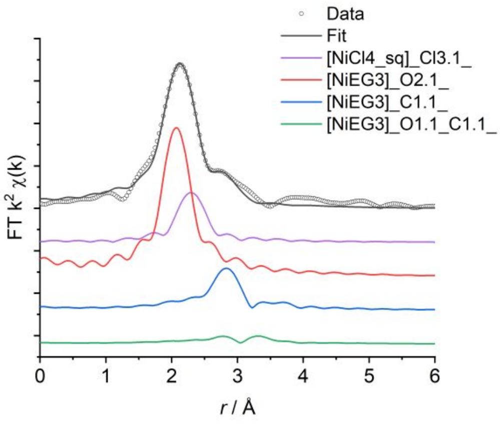

i) ChCl:2EG at 90 °C (k²-space)

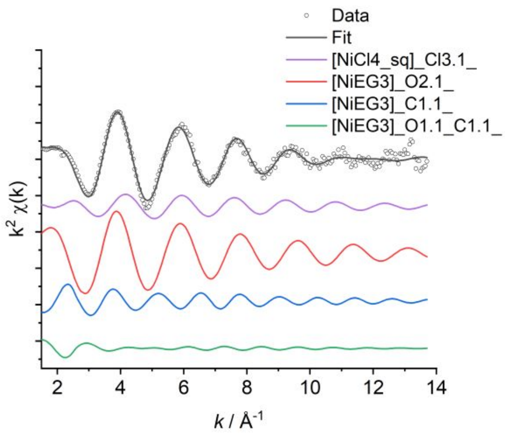

j) ChCl:2EG at 90 °C (r-space)

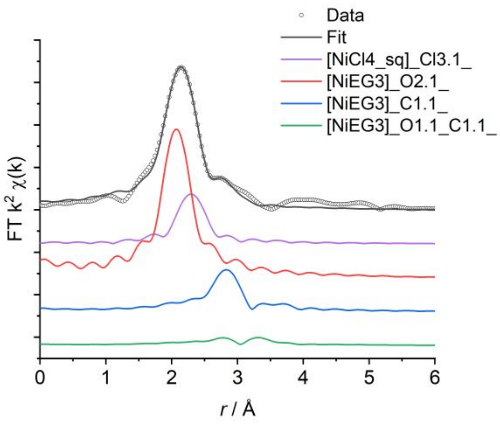

k) ChCl:2EG at 100 °C (k²-space)

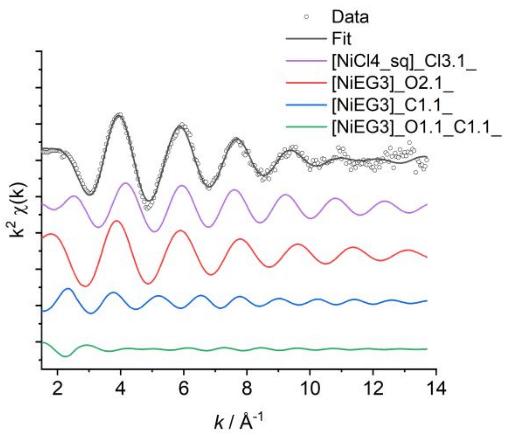

l) ChCl:2EG at 100 °C (r-space)

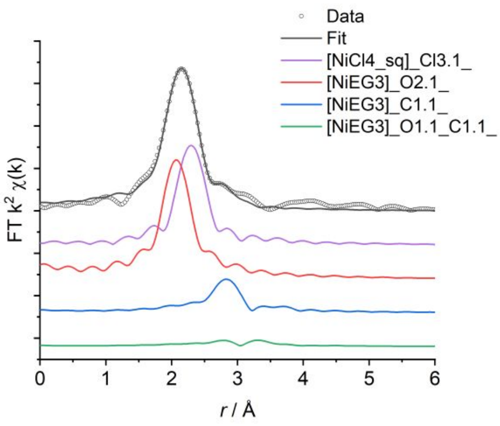

Figure S3 (cont.): k^2-weighted EXAFS (left) and Fourier transform (right) of 0.1 mol dm^{-3} $NiCl_2·6H_2O$ in ChCl:2EG, at temperatures between 23 and 120 °C. Data are dots, fits are lines. Model scattering paths used during fitting offset for clarity.

m) ChCl:2EG at 110 °C (k²-space)

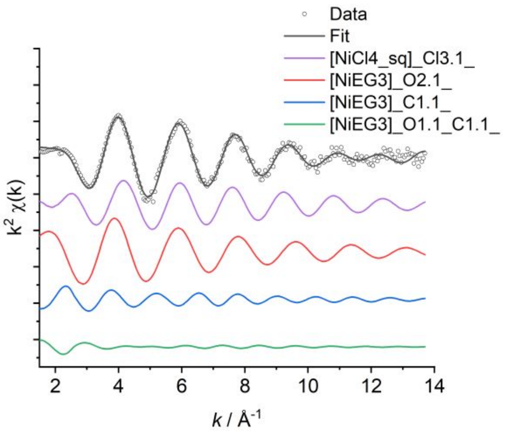

n) ChCl:2EG at 110 °C (r-space)

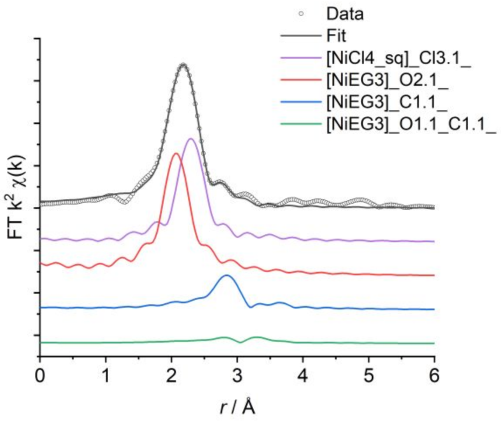

o) ChCl:2EG at 120 °C (k²-space)

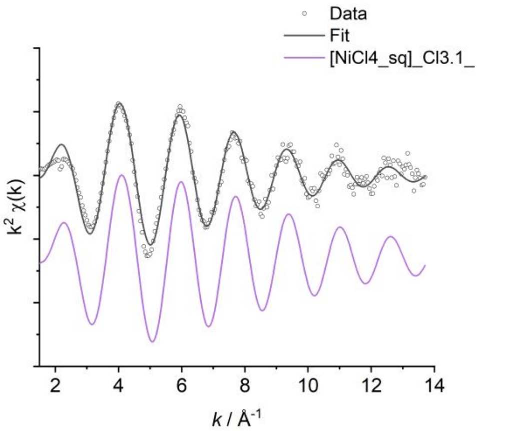

p) ChCl:2EG at 120 °C (r-space)

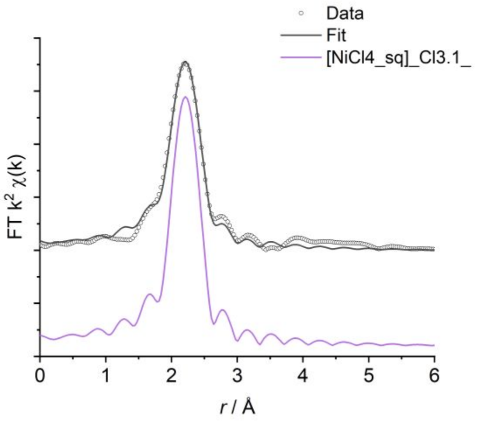

Figure S3 (cont.): k^2-weighted EXAFS (left) and Fourier transform (right) of 0.1 mol dm^{-3} $NiCl_2{\cdot}6H_2O$ in ChCl:2EG, at temperatures between 23 and 120 °C. Data are dots, fits are lines. Model scattering paths used during fitting offset for clarity.

a) ChCl:2U at 23 °C (k^2-space)

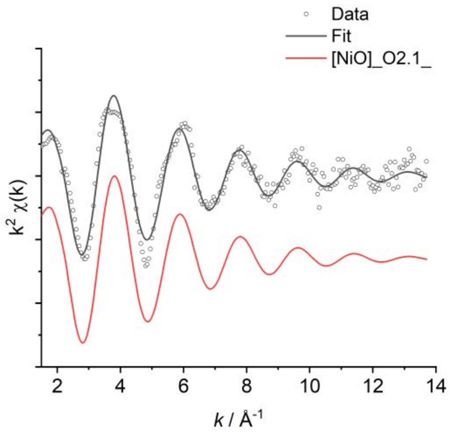

b) ChCl:2U at 23 °C (r-space)

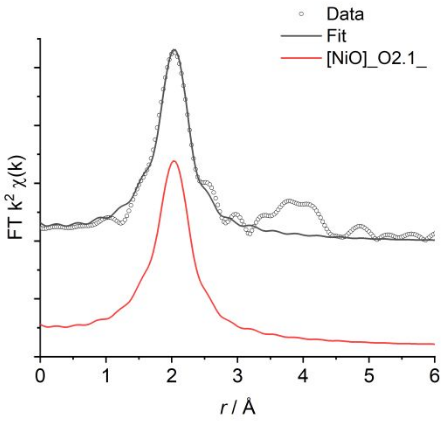

c) ChCl:2U at 50 °C (k^2-space)

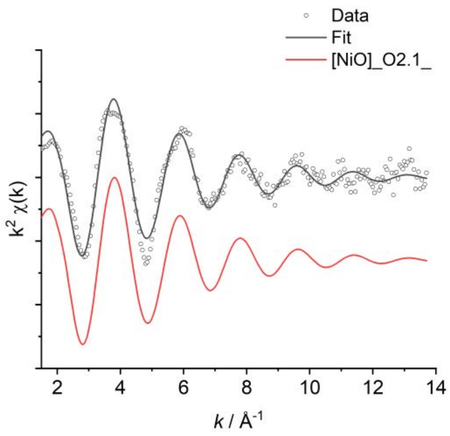

d) ChCl:2U at 50 °C (r-space)

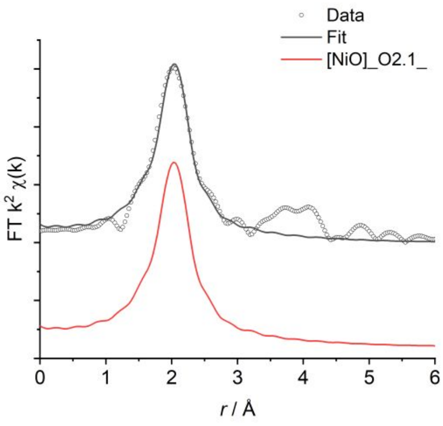

e) ChCl:2U at 70 °C (k^2-space)

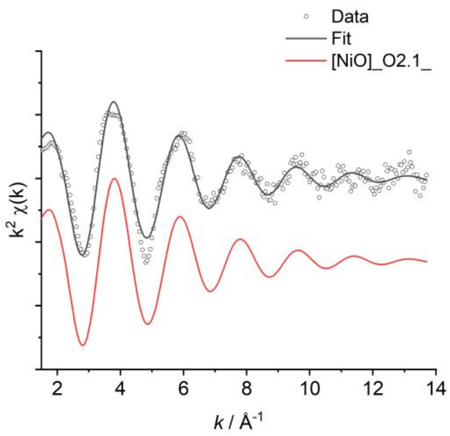

f) ChCl:2U at 70 °C (r-space)

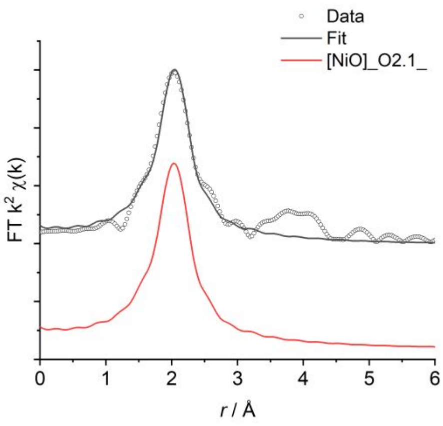

Figure S4: k^2-weighted EXAFS (left) and Fourier transform (right) of 0.1 mol dm^{-3} $NiCl_2{\cdot}6H_2O$ in ChCl:2U, at temperatures between 23 and 110 °C. Data are dots, fits are lines. Model scattering paths used during fitting offset for clarity.

g) ChCl:2U at 80 °C (k^2-space)

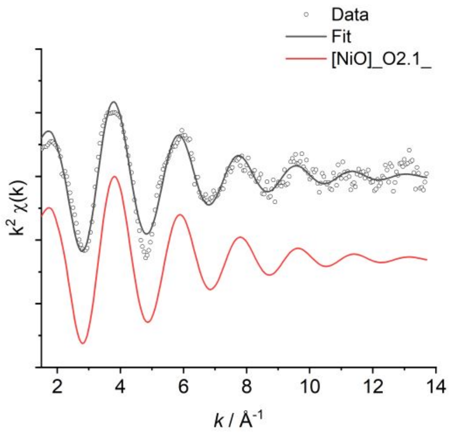

h) ChCl:2U at 80 °C (r-space)

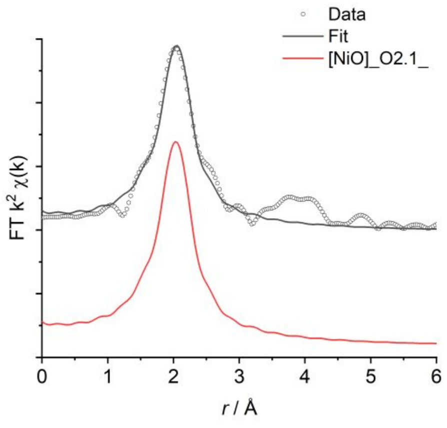

i) ChCl:2U at 90 °C (k^2-space)

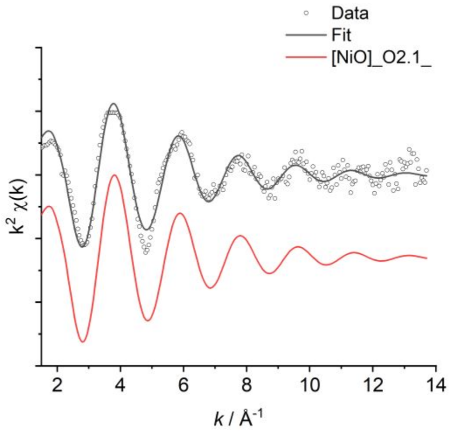

j) ChCl:2U at 90 °C (r-space)

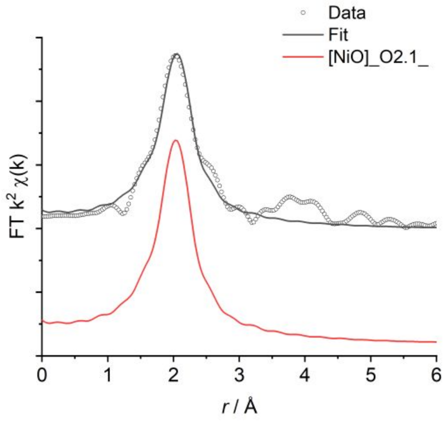

k) ChCl:2U at 100 °C (k^2-space)

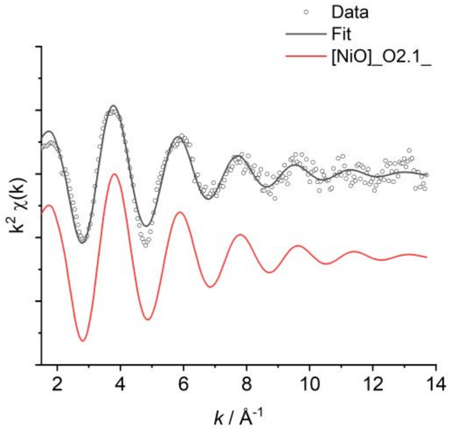

l) ChCl:2U at 100 °C (r-space)

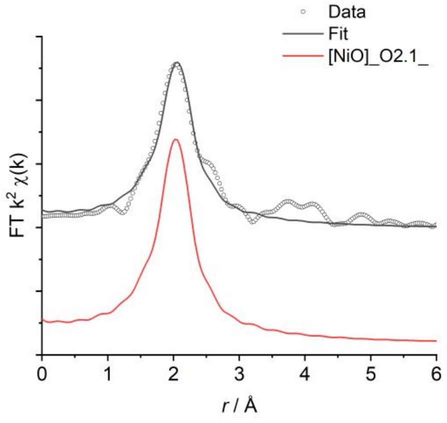

Figure S4 (cont.): k^2-weighted EXAFS (left) and Fourier transform (right) of 0.1 mol dm^{-3} $NiCl_2{\cdot}6H_2O$ in ChCl:2U, at temperatures between 23 and 110 °C. Data are dots, fits are lines. Model scattering paths used during fitting offset for clarity.

m) ChCl:2U at 110 °C (k^2-space)

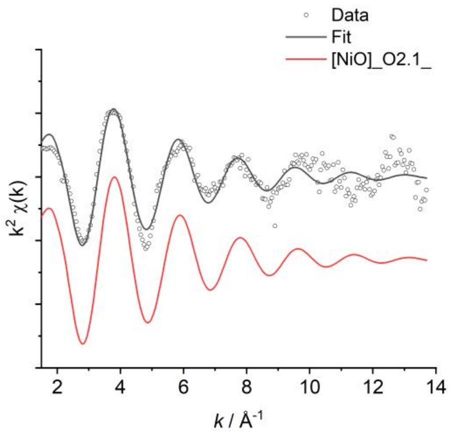

n) ChCl:2U at 110 °C (r-space)

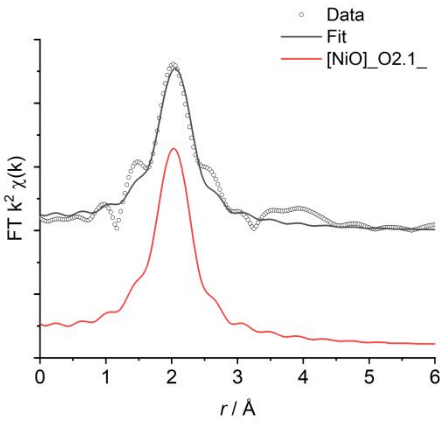

Figure S4 (cont.): k^2-weighted EXAFS (left) and Fourier transform (right) of 0.1 mol dm^{-3} $NiCl_2 \cdot 6H_2O$ in ChCl:2U, at temperatures between 23 and 110 °C. Data are dots, fits are lines. Model scattering paths used during fitting offset for clarity.

a)

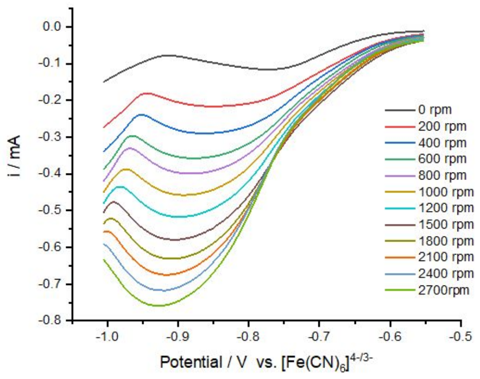

b)

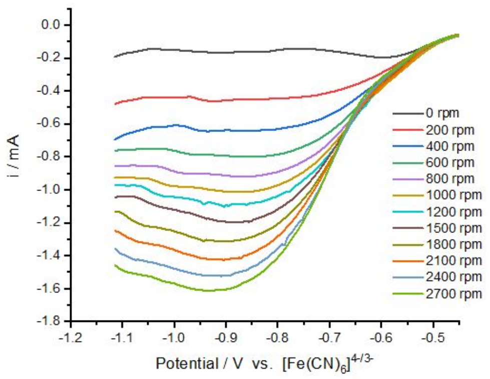

Figure S5: Rotating disc electrode voltammetry for 50 mM $NiCl_2 \cdot 6H_2O$ in ChCl:2EG at a) 80 °C, and b) 120 °C, using a 3 mm diameter Au disc working electrode, measured at a scan rate of 10 mV s^{-1}. The counter electrode was a Pt flag, and scans are referenced against the $[Fe(CN)_6]^{3-/4-}$ couple as an internal standard.

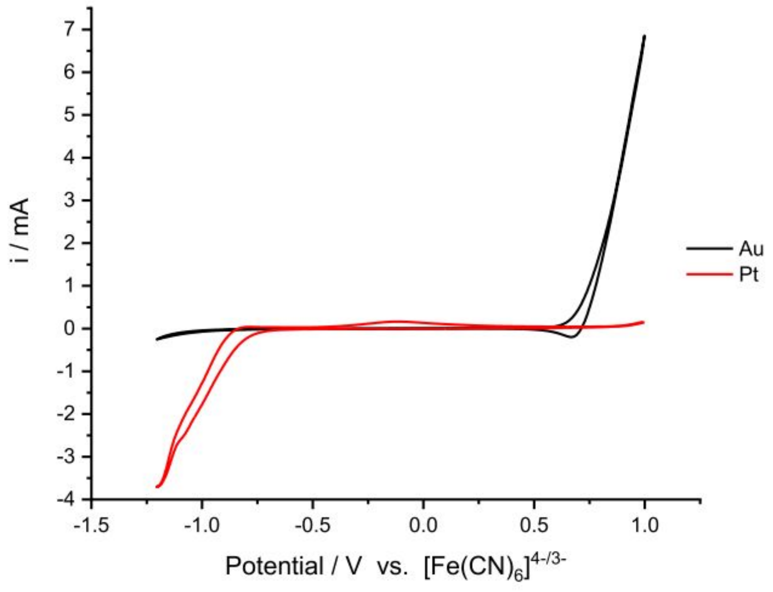

Figure S6: Cyclic voltammogram of ChCl:2EG at 120°C, using 3 mm diameter Pt and Au disc electrodes, at a scan rate measured at a scan rate of 10 mV s^{-1}. The counter electrode was a Pt flag, and scans are referenced against the $[Fe(CN)_6]^{3-/4-}$ couple as an internal standard.

References

1. B. Ravel and M. Newville, *J. Synchrotron Rad.*, 2005, **12**, 537-541.
2. Y. Tian, B. Etschmann, W. Liu, S. Borg, Y. Mei, D. Testemale, B. O'Neill, N. Rae, D. M. Sherman, Y. Ngothai, B. Johannessen, C. Glover and J. Brugger, *Chem. Geol.*, 2012, **334**, 345-363.
